# New TRUS Techniques and Imaging Features of PI-RADS 4 or 5: Influence on Tumor Targeting

**DOI:** 10.3389/fonc.2021.608409

**Published:** 2021-06-09

**Authors:** Amy Inji Chang, Byung Kwan Park

**Affiliations:** Department of Radiology, Samsung Medical Center, Sungkyunkwan University School of Medicine, Seoul, South Korea

**Keywords:** prostate adenocarcinoma, transrectal ultrasound, cognitive biopsy, fusion biopsy, magnetic resonance imaging

## Abstract

**Purpose:**

To determine if the new transrectal ultrasound (TRUS) techniques and imaging features contribute to targeting Prostate Imaging and Reporting and Data System (PI-RADS) 4 or 5.

**Materials and Methods:**

Between December 2018 and February 2020, 115 men underwent cognitive biopsy by radiologist A, who was familiar with the new TRUS findings and biopsy techniques. During the same period, 179 men underwent magnetic resonance imaging–TRUS image fusion or cognitive biopsy by radiologist B, who was unfamiliar with the new biopsy techniques. Prior to biopsy, both radiologists knew MRI findings such as the location, size, and shape of PI-RADS 4 or 5. We recorded how many target biopsies were performed without systematic biopsy and how many of these detected higher Gleason score (GS) than those detected by systematic biopsy. The numbers of biopsy cores were also obtained. Fisher Exact or Mann–Whitney test was used for statistical analysis.

**Results:**

For PI-RADS 4, target biopsy alone was performed in 0% (0/84) by radiologist A and 0.8% (1/127) by radiologist B (p>0.9999). Target biopsy yielded higher GSs in 57.7% (30/52) by radiologist A and 29.5% (23/78) by radiologist B (p = 0.0019). For PI-RADS 5, target biopsy alone was performed in 29.0% (9/31) by radiologist A and 1.9% (1/52) by radiologist B (p = 0.0004). Target biopsy yielded higher GSs in 50.0% (14/28) by radiologist A and 18.2% (8/44) by radiologist B (p = 0.0079). Radiologist A sampled fewer biopsy cores than radiologist B (p = 0.0008 and 0.0023 for PI-RADS 4 and 5), respectively.

**Conclusions:**

PI-RADS 4 or 5 can be more precisely targeted if the new TRUS biopsy techniques are applied.

## Introduction

Prostate Imaging and Reporting and Data System (PI-RADS) 4 or 5 should be biopsied because these lesions have a much higher incidence of being confirmed as significant cancer than do lesions with PI-RADS 3 or less (1–5). When PI-RADS 4 or 5 is detected on magnetic resonance imaging (MRI), transrectal ultrasound (TRUS)-guided cognitive or MRI-TRUS fusion biopsy is performed to detect significant cancers.

Recently, several investigators reported the new TRUS features of peripheral or transition PI-RADS 4 or 5 lesions (6–11). They also introduced new TRUS techniques, such as how to choose the imaging sequence, control image contrast, compress the prostate, and localize a tumor (6–11). However, they did not determine whether being familiar with the new TRUS techniques and imaging features influenced on tumor targeting. Still, the utility of the new biopsy techniques in targeting cancer remains unclear.

Accordingly, we hypothesized that TRUS-guided cognitive biopsy using the new TRUS techniques and features would improve targeting PI-RADS 4 or 5. The study aim was to determine the effect of operator familiarity with the new biopsy techniques on tumor targeting.

## Materials and Methods

### Patient Selection

**Figure 1:**
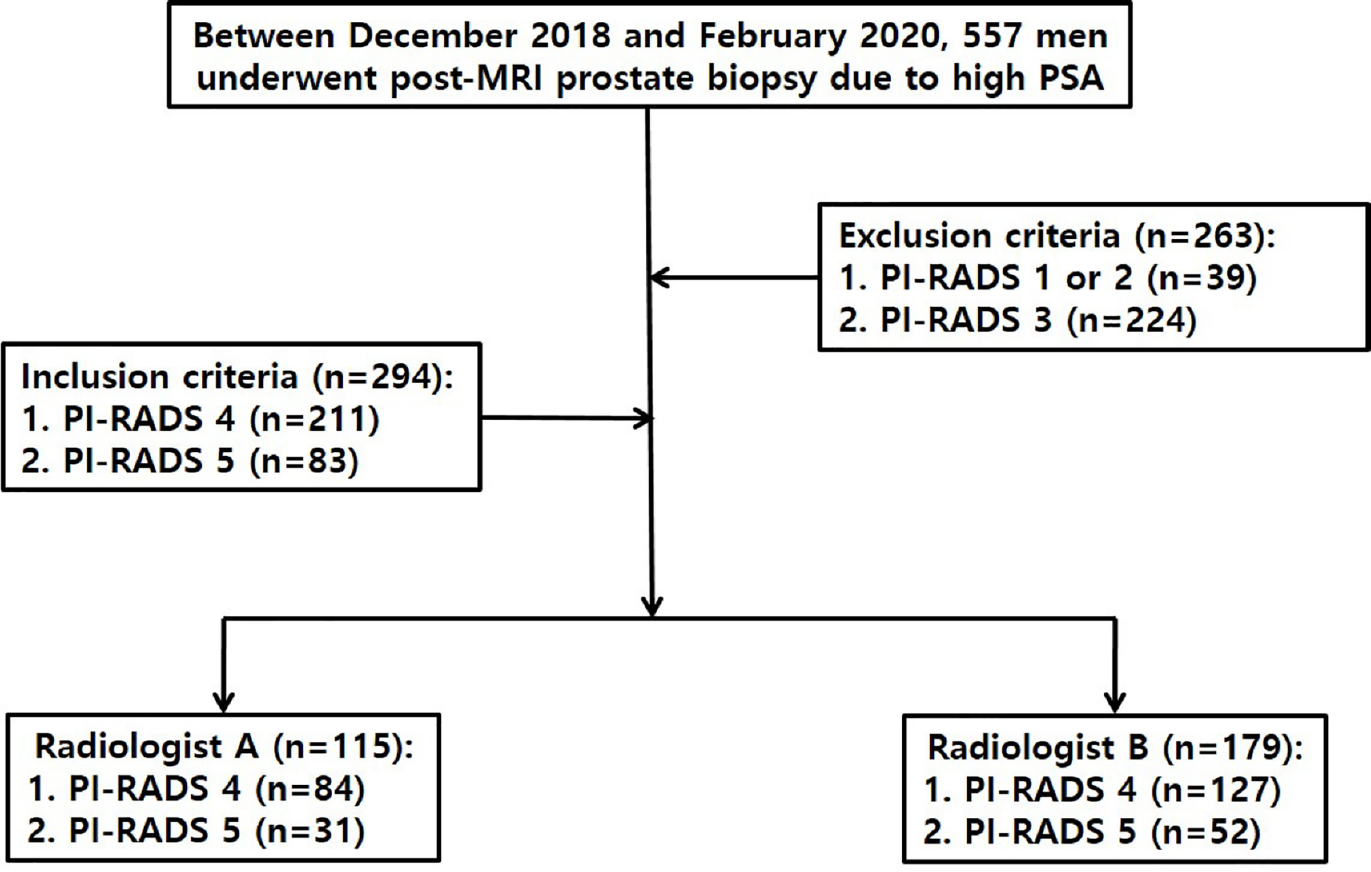
Flow diagram illustrating how to select study population.

Between December 2018 and February 2020, 557 men underwent TRUS-guided cognitive or MRI-TRUS fusion biopsy because of high (2.5 ng/ml or greater) prostate-specific antigen (PSA) after MRI was performed prior to all biopsies (**Figure 1**). Of these patients, 263 were excluded according to the following criteria: PI-RADS 1–2 (n = 39), and 3 (n = 224). The remaining 294 were included because an index lesion was categorized as PI-RADS 4 (n = 211) or 5 (n = 83) on pre-biopsy MRI. Radiologist A performed TRUS-guided cognitive biopsy in 115 men, whose index lesion was PI-RADS 4 in 84 men and 5 in 31 men on pre-biopsy MRI. Radiologist B performed MRI-TRUS fusion biopsy (n = 176) or TRUS-guided cognitive biopsy (n = 3) in 179 men, whose index lesion was PI-RADS 4 in 127 men and 5 in 52 men on pre-biopsy MRI. Each radiologist, who interpreted pre-biopsy MR images in a patient, was supposed to perform a biopsy in the same patient.

### Biopsy Techniques

**Figure 2:**
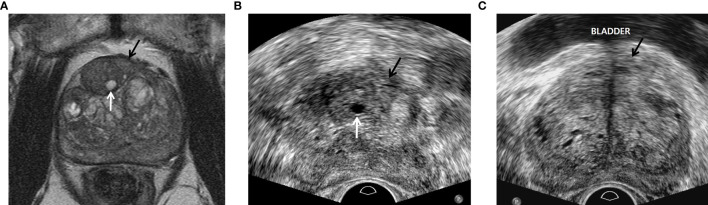
A 77-year-old man with PSA of 9.57 ng/ml. **(A)** T2-weighted axial magnetic resonance image showing a PI-RADS 4 transition mass (black arrow) in the left base. The tumor is contacting the anterior capsule. The white arrow indicates a small cyst in the neighboring hyperplastic nodule. **(B)** Transverse TRUS image scanned by radiologist A showing a slightly hyperechoic mass (black arrow) in the left mid-gland. The tumor was targeted with four cores of which three were GS 6 and one was negative. Sextant systematic biopsy was also performed, but all of the cores were negative. The white arrow indicates a small cyst in the neighboring hyperplastic nodule, which is the same imaging feature seen on T2-weighted MR image. This TRUS sequence is fundamental imaging with low dynamic range, resulting in good contrast between the PI-RADS 4 lesion and normal tissue. **(C)** Transverse TRUS image, which was scanned by radiologist B two months before beginning our study period, shows a slightly hypoechoic mass (black arrow) in the left base contacting the bladder base. The tumor was targeted with three cores of which all were negative. Systematic biopsy was also performed with 10 cores of which all were negative. Radiologist B did not detect the neighboring hyperplastic nodule with a small cyst that was seen on the T2-weighted MR image. This TRUS sequence is harmonic imaging with high dynamic range, subsequently leading to poor tissue contrast.

Radiologists A and B had already performed TRUS-guided cognitive or MRI-TRUS fusion biopsy in more than 500 men each before this study period began. Radiologist A alone was familiar with the following TRUS techniques and imaging features (6–9): First, fundamental imaging was performed instead of harmonic imaging (**Figure 2**). Second, the TRUS dynamic range was kept to 50 or less (**Figure 2**). Third, the prostate was not compressed until PI-RADS 4 or 5 was detected. Fourth, a TRUS lesion appeared more superiorly than an MRI lesion as it was nearer to the posterior capsule. Fifth, a TRUS lesion appeared more inferiorly than an MRI lesion as it was nearer to the anterior capsule (**Figure 2**). Sixth, peripheral and transition PI-RADS 4 or 5 looked hypoechoic and hyperechoic relative to neighboring normal tissue, respectively (**Figure 2**). Finally, PI-RADS 5 tended to be more hypoechoic or hyperechoic than PI-RADS 4 or less. However, radiologist B was not familiar with these new TRUS techniques and features for targeting PI-RADS 4 or 5 (**Figure 2**).

Both of two radiologists were fully aware of the location, size, and shape of an index lesion on MRI. Radiologist A, who performed cognitive biopsy alone, already knew how to use the new TRUS features and techniques. Radiologist B, who performed MRI-TRUS fusion biopsy or cognitive biopsy, did not know it.

Radiologists A and B used one of the following ultrasonography (US) scanners: EPIC (Philips Health Care, Bothell, WA, USA), IU22 (Philips Health Care), or Aplio 500 (Toshiba Medical System, Japan). Radiologist A did not use MRI-TRUS fusion software, but radiologist B used Fly Thru and Smart Fusion (Toshiba Medical System) for MRI-TRUS fusion imaging.

### Data Analysis

Patients’ ages, PSA levels, tumor sizes, and tumor locations were compared between the groups undergoing the biopsies performed by radiologist A or B. The sizes of PI-RADS 4 and 5 were measured on diffusion-weighted MR images for peripheral tumors or on T2-weighted MR images for transition tumors. The tumor locations were recorded as peripheral-to-transition ratios in each group.

The numbers of target and systematic biopsies were compared to identify how many patients underwent target biopsy alone without relying on systematic biopsy. Systematic biopsy was not performed only when the radiologists ensured that an index lesion was precisely targeted. However, systematic biopsy alone was performed only when an index lesion was invisible on TRUS or when software fusion of MRI-TRUS images was too poor to target an index lesion.

Target and systematic biopsies were compared in patients with cancer-proven PI-RADS 4 or 5 in terms of Gleason score (GS) to identify how many targeted biopsies were superior, equal, and inferior to systematic biopsies. These data were also compared between radiologists A and B to determine which biopsy technique was more accurate in targeting an index lesion.

The numbers of target or systematic cores that each radiologist sampled in each patient with cancer-proven PI-RADS 4 or 5 were compared to determine which radiologist took fewer cores. Positive to negative core ratios (PNCRs) were compared between radiologists A and B to assess how efficiently cancer was detected with the target or systematic cores.

The cancer detection rates (CDRs) were compared between radiologists A and B. The CDR was calculated as the number of cancer cases divided by the total number of cases. The significant CDR was calculated as the number of GS 7 or higher cases divided by the total number of cases.

### Standard Reference

The standard reference was histological examination of the prostate biopsies that were performed by radiologists A and B. Significant cancer was defined as a tumor with a GS ≥ 7 (3 + 4).

### Statistical Analysis

Mann–Whitney test was performed to compare the patients’ ages, PSA levels, tumor sizes, and biopsy cores between the PI-RADS 4 or 5 group. Fisher exact test was also performed to compare tumor locations, target-to-systematic biopsy ratios, PNCRs, and CDRs. Commercial software (PASW Statistics, version 20.0; Chicago, IL, USA) was used for statistical analysis. A two-sided *p* value of less than 0.05 was considered statistical significance.

## Results

The median PSA levels of the PI-RADS 4 groups biopsied by radiologists A and B were 4.58 ng/ml (2.50–16.44 ng/ml) and 5.81 ng/ml (2.50–46.70 ng/ml), respectively (**Table 1**) (p = 0.0001). However, there was no significant difference between the PI-RADS 4 or 5 groups in terms of the other patients’ demographics (**Table 1**) (p = 0.4154–0.999).

**Table 1:** Patients’ demographics.

	PI-RADS 4 (n = 211)		P values	PI-RADS 5 (n = 83)		P values
	RA group (n = 84)	RB group (n = 127)		RA group (n = 31)	RB group (n = 52)	
Age (years)	67.0 (43.0–80.0)	65.0 (42.0–83.0)	0.0763	67.0 (55.0–98.0)	68.5 (49.0–84.0)	0.5947
PSA (ng/ml)	4.58 (2.50–16.44)	5.81 (2.50–46.70)	0.0001	8.69 (3.34–747.30)	9.22 (2.50–84.6)	0.8323
Tumor size (mm)	9.5 (4.0–14.5)	10.0 (3.7–13.8)	0.8402	19.0 (15.7–58.0)	21.0 (15.0–42.0)	0.4154
PZ to TZ ratio	69:15	105:22	>0.9999	17:14	26:26	0.8207

PI-RADS, Prostate Imaging and Reporting and Data System; RA, Radiologist A; RB, Radiologist B; PSA, Prostate-specific antigen; PZ, Peripheral Zone; TZ, Transition Zone; All data except lesion location are shown as the median (range).

For the PI-RADS 4 tumors, radiologists A performed target and systematic biopsies in all (100%, 84/84) patients, and radiologist B performed the combination biopsy in 126 (99.2%, 126/127) patients except one who had target biopsy alone (p > 0.9999). The overall CDRs were 64.3% (54/84) for radiologist A and 61.4% (78/127) for radiologist B (p = 0.7715). The significant CDRs were 40.5% (34/84) for radiologist A and 43.3% (55/127) for radiologist B (p = 0.2141).

For the PI-RAD 5 tumors, however, radiologists A and B performed target biopsy alone in nine (29.0%, 9/31) patients and in one (1.9%, 1/52) patient, respectively (p = 0.0004). The overall CDRs were 90.3% (28/31) for radiologist A and 84.6% (44/52) for radiologist B (p = 0.5249). The significant CDRs were 83.9% (26/31) for radiologist A and 76.9% (40/52) for radiologist B (p = 0.5776).

For the cancer-proven PI-RAD 4 cases, target biopsy was superior to systematic biopsy in 57.7% (30/52) by radiologist A and in 29.5% (23/78) by radiologist B (**Table 2**) (p = 0.0019). Target biopsy was equal to systematic biopsy in 19.2% (10/52) by radiologist A and in 44.9 (35/78) by radiologist B (**Table 2**) (p = 0.0027). However, there was no significant difference between radiologists A and B in the number of patients in which the target biopsy was inferior to systematic biopsy (**Table 2**) (p = 0.8365). The median numbers of target and systematic cores were 11.0 (8.0–12.0) for radiologist A and 12.0 (8.0–14.0) for radiologist B (p = 0.0008). The median numbers of target cores were 5.0 (2.0–6.0) for radiologist A and 2.0 (2.0–5.0) for radiologist B (p < 0.0001), whereas those of the systematic cores were 6.0 (2.0–6.0) for radiologist A and 10.0 (5.0–10.0) for radiologist B (p < 0.0001). The PNCRs were 248:330 for radiologist A and 338:561 for radiologist B (p = 0.0439).

**Table 2:** Comparison of Gleason scores from target and systematic biopsies.

	Cancer-proven PI-RADS 4 (n = 130)		P values	Cancer-proven PI-RADS 5 (n = 72)		P values
	RA group (n = 52)	RB group (n = 78)		RA group (n = 28)	RB group (n = 44)	
T > S (%)	57.7 (30/52)	29.5 (23/78)	0.0019	50.0 (14/28)	18.2 (8/44)	0.0079
T = S (%)	19.2 (10/52)	44.9 (35/78)	0.0027	39.3 (11/28)	63.6 (28/44)	0.0545
T < S (%)	23.1 (12/52)	25.6 (20/78)	0.8365	10.7 (3/28)	18.2 (8/44)	0.5108

PI-RADS, Prostate Imaging and Reporting and Data System; RA, Radiologist A; RB, Radiologist B; T > S, T = S, and T < S indicates that the Gleason scores of the target biopsy were superior, equal, and inferior, respectively, to those of the systematic biopsy.

For the cancer-proven PI-RADS 5 cases, target biopsy was superior to systematic biopsy in 50.0% (14/28) by radiologist A and in 18.2% (8/44) by radiologist B (**Table 2**) (p = 0.0079). However, there was no significant difference between radiologists A and B in the number of patients for whom target biopsy was equal or inferior to systematic biopsy (**Table 2**) (p = 0.0545 or 0.5108). The median numbers of target and systematic cores were 10.0 (3.0–13.0) for radiologist A and 11.0 (7.0–13.0) for radiologist B (p = 0.0023). The median numbers of target cores were 5.0 (3.0–8.0) for radiologist A and 3.0 (0.0–7.0) for radiologist B (p < 0.0001), whereas those of the systematic biopsy were 6.0 (0.0–6.0) for radiologist A and 9.0 (0.0–11.0) for radiologist B (p < 0.0001). The PNCRs were 149:84 for radiologist A and 250:229 for radiologist B (p = 0.0037).

Among the PI-RAD 4 groups, the overall and significant CDRs of target biopsies performed by radiologist A were 54.8% (46/84) and 35.7% (30/84), whereas those of target biopsies performed by radiologist B were 51.2% (65/127) and 34.6% (44/127), respectively. Among the PI-RAD 5 groups, the overall and significant CDRs of target biopsies performed by radiologist A were 87.1% (27/31) and 80.6% (25/31), whereas those of target biopsies performed by radiologist B were 82.7% (43/52) and 73.1% (38/52), respectively. The p values of overall CDRs in the PI-RADS 4 and 5 were 0.6733 and 0.7583, and those of significant CDRs in the PI-RADS 4 and 5 were 0.8838 and 0.5968, respectively.

## Discussion

Our results showed that a greater number of target biopsies yielded higher GSs than did systematic biopsies by radiologist A who was familiar with the new TRUS techniques and features of PI-RADS 4 or 5. For this reason, a greater number of men with PI-RADS 5 underwent target biopsy alone by radiologist A than by radiologist B who did not know the new TRUS techniques and features of PI-RADS 4 or 5. Moreover, radiologist A was able to perform biopsies with fewer cores because of higher PNCRs than was radiologist B.

Recent US scanners recommend the use of harmonic imaging rather than fundamental imaging because the former provides better axial and lateral resolutions than does the latter (12–14). However, increasing tissue resolution inevitably results in decreasing tissue contrast between prostate cancer and normal tissue. Prostate cancer that is located near the transducer can be depicted with poor contrast because of the lack of harmonics in the incident ultrasound wave (15). Therefore, radiologist A did not use harmonic imaging but rather fundamental imaging. Moreover, he maintained lower dynamic range to enhance tissue contrast by sharpening the tumor edges (16).

Tumor locations appear so different between MRI and TRUS because of the different scan axes. Axial MRI images are scanned along the perpendicular axis of the prostate urethra, whereas axial TRUS images are scanned along the oblique axis of the prostate urethra (6, 7, 10, 17). For this reason, when PI-RADS 4 or 5 is closer to the anterior capsule, the tumor seems to be located more inferiorly on TRUS than on MRI. Given that PI-RADS 4 or 5 is closer to the posterior capsule, the tumor seems to be located more superiorly on TRUS than on MRI. Currently, urologists or radiologists who perform TRUS-guided cognitive biopsy try to find an index tumor at the same level that is seen on MRI. Therefore, their targeting is likely to be so poor that significant cancer cannot be sampled precisely. Another technical tip for achieving good lesion depiction is to not compress the prostate until PI-RADS 4 or 5 is detected. Compression deforms the shape of the PI-RADS 4 or 5 but also obscures a small tumor because it is frequently embedded in the normal tissue (6, 9, 10, 17).

Urologists or radiologists are used to know that the TRUS features of prostate cancer are hypoechoic regardless of lesion location. Peripheral cancer was hypoechoic compared with the neighboring normal tissue, but transition cancer was hyperechoic compared with the neighboring hyperplastic nodules. Several papers have reported that some prostate cancers are hyperechoic compared with adjacent tissue (18–20). The incidence of hyperechoic tumors is reported as high as 40% (20). Unfortunately, the researchers did not demonstrate that these cancers arise from transition cancers. However, Park et al. have showed that transition cancer tends to be more hyperechoic than does peripheral cancer (6).

Many papers have reported that significant CDRs range from 22.1% to 78.0% for PI-RADS 4 and 72.4% to 90.7% for PI-RADS 5 (3, 4, 21–23). Radiologist A achieved a relatively lower significant CDR (40.5%) for PI-RADS 4, although the new TRUS techniques and features were applied for biopsy. The median size of PI-RADS 4 was less than 10 mm, which subsequently led to a decreasing significant CDR (9). Moreover, the median PSA levels were lower in the PI-RADS 4 patients who were biopsied by radiologist A. However, radiologist A achieved a relatively higher significant CDR (83.9%) for PI-RADS 5 because there was no difference between the biopsy groups in the lesion sizes and PSAs.

Radiologist A could not avoid performing a systematic biopsy for PI-RADS 4 even though the tumor was clearly seen on TRUS. The main reason was that PI-RADS 4 was smaller than PI-RADS 5, and thus he could not ensure that it was targeted 100%. Moreover, the systematic biopsy achieved higher GSs than those of the target biopsy in a small number of PI-RADS 4 or 5 tumors. Because significant cancer can be detected in PI-RADS 3 or less, a systematic biopsy should be added to the target biopsy for PI-RADS 4. However, radiologist A omitted the systematic biopsy in a substantial number of PI-RADS 5 tumors, especially for men who did not stop taking aspirin because of coronary artery stent. Therefore, the new techniques of TRUS biopsy may contribute not only to a decreasing number of biopsy cores but also to a reduction in the complication rate (6).

Indeed, the purpose of our investigation was not to compare cognitive fusion and image fusion biopsy in terms of tumor targeting. Whether or not a biopsy operator knows the new TRUS techniques and imaging features can influence tumor targeting. Radiologist A knew it, but radiologist B did not. Radiologist B as well as radiologist A also performed cognitive biopsy although the number of cognitive biopsies was small. Because he did not know the new TRUS techniques and imaging features, he was not able to precisely detect an index tumor, which is shown in **Figure 2**. Besides, the image quality of his TRUS was inferior to that of radiologist A’s TRUS. In other words, the new TRUS techniques and imaging features help to improve image quality, cancer detection, and tumor targeting regardless of biopsy types (cognitive fusion or image fusion) if a biopsy operator is familiar with them. Many readers frequently misunderstand that our study was to compare cognitive fusion and image fusion biopsies in terms of tumor targeting.

Our current policy of TRUS biopsy is that systematic biopsy is routinely performed after an index tumor is targeted because it can detect additional significant cancers where PI-RADS 1 or 2 were diagnosed (11, 24). PI-RADS 1-2 does not indicate that CDR is 0% (23). Radiologist A achieved higher overall and significant CDRs of target biopsies than radiologist B even if the PSA levels and tumor sizes of PI-RADS 4 and 5 groups, which radiologist A performed biopsies, were lower/smaller than those of PI-RADS 4 and 5 groups, which radiologist A performed biopsies. Unfortunately, there was no significant difference between the groups in terms of CDR due to small number of biopsy cases. However, target biopsies of radiologist A are so likely to provide much lower underestimation of GS compared to prostatectomies because of good tumor targeting. GS underestimation frequently induces under-treatment of prostate cancer (25).

Our study had some limitations. First, the standard reference was not a prostatectomy but rather a biopsy examination because a substantial number of cancer-proven patients underwent active surveillance, hormone therapy, radiation therapy, or chemotherapy. Therefore, the biopsy GS did not correlate with the surgical GS in all patients. Second, the targeting of PI-RADS 2 or 3 between radiologists A and B were not compared. Lower PI-RADS scores appear to be more difficult to detect with TRUS. Further investigation is necessary to assess the utility of the new biopsy techniques in targeting lower PI-RADS scores. Third, the number of PI-RADS 4 or 5 lesions was relatively smaller for radiologist A than for radiologist B. However, the number of PI-RADS 3 lesions, which were excluded from the current study, was much larger for radiologist A than for radiologist B. Our urologists had previously recognized that the new biopsy techniques were better than the conventional MRI-TRUS fusion or cognitive biopsy techniques for tumor targeting. Thus, they transferred a larger number of PI-RADS 3 cases to radiologist A because this category required more precise targeting. Fourth, the same biopsy modality was not used to compare radiologists A and B in terms of tumor targeting. Recent meta-analysis reported that there is no difference between MRI-TRUS cognitive fusion and MRI-TRUS image fusion biopsies in terms of cancer detection rate (26). Our study showed no significant difference between A and B in terms of cancer detection rate, either. However, whether or not radiologists or urologists are familiar with the new TRUS findings and techniques influence the tumor targeting significantly regardless of types of biopsies that they perform. For more than 10 years, radiologists A and B have performed MRI-TRUS cognitive fusion or MRI-TRUS image fusion biopsies. However, radiologist A discovered new TRUS findings and techniques, leading to improve tumor targeting four years ago. Since then, he has performed only MRI-TRUS cognitive fusion biopsy definitely based on the new TRUS findings and techniques in order to improve targeting an index lesion. In contrast, radiologist B insisted that MRI-TRUS image fusion biopsy is more reliable to tumor targeting. Therefore, this was the background of our research that being familiar with the new TRUS findings and techniques helps radiologists or urologists to have more precise tumor targeting regardless of types of biopsies. Our results and figures showed that there was no difference between A and B in terms of significant or insignificant cancer detection rate. Radiologist A demonstrated that the Gleason scores of target biopsies were significantly higher than those of systematic biopsies. Accordingly, he was able to omit the systematic biopsy in many cases or reduce the number of biopsy cores. In contrast, radiologists B showed that the Gleason scores of target biopsy were not different from those of systematic biopsy. Accordingly, he was not able to omit the systematic biopsy, resulting in increasing the number of biopsy cores and complication rates. If radiologist B had been familiar to the new TRUS findings and techniques, his tumor targeting could have been more precise. Recently, our urologists have transferred only to the radiologist A so many patients who cannot stop aspirin medication due to cardiovascular diseases because they ask him to perform a target biopsy alone in them. When we carefully see the **Figure 2** images, radiologist A detected a true lesion in the mid-gland and cancer was detected. However, radiologist B was not able to detect a true lesion because he did not know the new TRUS findings and techniques, and his biopsy results were negative. Fifth, there was different demographics between PI-RADS 4 or 5 groups. The PSA levels of PI-RADS 4 and 5 groups, who radiologist A biopsied, were lower than those of PI-RADS 4 and 5 groups, who radiologist B biopsied. Besides, the tumor sizes of PI-RADS 4 and 5 groups, who radiologist A biopsied, were smaller than those of PI-RADS 4 and 5 groups, who radiologist B biopsied. Generally, as PSA levels and tumor sizes increase, cancer detection rates (CDRs) also increase. Nonetheless, the CDRs of radiologist A were higher than those of radiologists B. Therefore, we do not think that the different PSA levels or tumor sizes had influence on tumor targeting. Sixth, we did not perform inter-reader agreement between radiologist A and B in terms of MRI interpretation. We can indirectly identify the disagreement. Overall and significant CDRs of PI-RADS 4 and 5 are similar to those of PI-RADS 4 and 5, which were reported by previous investigations (10, 23). Besides, PI-RADS 4 and 5 provide lower inter-reader agreement between radiologists compared to PI-RADS 2 or 3. We do not think that disagreement will not be high. Finally, our study was a retrospective design. Accordingly, we cannot completely exclude a selection bias in including study population. MRI-TRUS fusion or cognitive biopsy does not fully obtain systemic puncture, and its accuracy or representativeness is still controversial.

## Conclusion

Radiologists or urologists can target PI-RADS 4 or 5 more precisely if they are familiar with the new TRUS techniques and imaging features. Systematic biopsy is considered as a routine procedure due to additional detection of prostate cancer. However, systematic biopsy might be omitted in PI-RAD 5 patients who have T3 or higher stage cancer, bleeding tendency, or anti-coagulant medication if the new TRUS techniques and imaging features are applied.

## Data Availability Statement

The raw data supporting the conclusions of this article will be made available by the authors, without undue reservation.

## Ethics Statement

The studies involving human participants were reviewed and approved by Samsung Medical Center, Institutional Review Board. Written informed consent for participation was not required for this study in accordance with the national legislation and the institutional requirements.

## Author Contributions

Conceptualization, BP. Methodology and software, BP. Formal analysis, AC. Investigation, BP. Resources, BP. Data curation, AC. Writing—original draft preparation, AC. Writing—review and editing, AC. and BP. Visualization, AC. Supervision, BP. Project administration, BP. All authors contributed to the article and approved the submitted version.

## Conflict of Interest

The authors declare that the research was conducted in the absence of any commercial or financial relationships that could be construed as a potential conflict of interest.
